# Metal–*N*-Heterocyclic Carbene Porous Organic Polymers as Efficient Bifunctional Water-Splitting Electrocatalysts

**DOI:** 10.3390/nano16120781

**Published:** 2026-06-21

**Authors:** Shasha Ma, Zhaobin Ye, Guang Shi, Jianyong Zhang

**Affiliations:** 1 MOE Laboratory of Polymeric Composite and Functional Materials, School of Materials Science and Engineering, Sun Yat-sen University, Guangzhou 510275, China; 2 School of Chemistry, South China Normal University, Guangzhou 510006, China

**Keywords:** porous organic polymers, metal–*N*-heterocyclic carbene, bifunctional catalysts, water-splitting

## Abstract

The design and manufacture of bifunctional electrocatalysts are of great significance in the electrolysis of water. Herein, porous organic polymers (POPs) of metal–*N*-heterocyclic carbene were synthesized from imidazolium borate ionic POPs and supported on a nickel foam surface (Pd–NHC/NF, Ag–NHC/NF, and Cu–NHC/NF). Among them, Pd–NHC/NF exhibited high activity for both hydrogen evolution reaction and oxygen evolution reaction. The oxygen evolution overpotential of Pd–NHC/NF was 245 mV with a Tafel slope of 83 mV dec^−1^; the hydrogen evolution overpotential was 139 mV with a Tafel slope of 94 mV dec^−1^ at 10 mA cm^−2^ in alkaline media. Additionally, the assembled Pd–NHC/NF||Pd–NHC/NF electrolyzer demonstrated excellent performance in electrocatalytic water-splitting, achieving a voltage of 1.55 V at 10 mA cm^−2^ and showing outstanding stability for over 90 h in the long-term test. The results highlighted the substantial capability of Pd–NHC as a bifunctional catalyst for electrocatalytic water-splitting.

## 1. Introduction

Hydrogen energy with high energy density and storability is regarded as a promising energy resource. Electrochemical water-splitting can be used for efficient and large-scale hydrogen generation [1,2]. Durable and efficient electrocatalysts are required to promote hydrogen evolution reaction (HER) and oxygen evolution reaction (OER) in large-scale water-splitting [3]. Presently, ruthenium oxide (RuO_2_) and iridium oxide (IrO_2_) are benchmark OER electrocatalysts, while platinum (Pt) is preferred for HER [4]. However, the high cost and scarce resources of precious metals limit their commercial-scale applications. Therefore, it is critical to control the amount of precious metals used.

Catalysts play an important role in reducing OER/HER overpotential and improving water electrolysis efficiency [5,6,7]. Transition metal-based oxides [8,9], metal complexes/coordination polymers [10,11], and layered double hydroxides [12] are widely used in OER. Meanwhile, phosphides [13], sulfides [14], and carbides [15] are promising HER catalysts. In order to improve the electrocatalytic activity and decomposition efficiency of water-splitting, a significant number of bifunctional electrocatalysts have been designed and synthesized. Various strategies have been adopted to optimize catalyst performance such as adjusting local electronic structures [16,17,18], enhancing charge transfer efficiency [19,20,21], and optimizing exposed active sites [22,23].

In recent years, nitrogen–heterocyclic carbenes (NHCs) have been widely used in the field of organometallic catalysis due to their simple synthesis, ease of handling, non-toxicity, robust stability, and tunable catalytic activity [24,25]. Because NHCs are strong σ-donors, metal complexes bound to NHCs typically exhibit inert M–NHC bonds, which enhance the overall stability [26,27]. To facilitate the separation and recovery, M–NHC catalysts have been immobilized on porous organic polymers (POPs) by virtue of their high surface area and tailor-made functional groups [28]. M–NHC catalysts have been employed to catalyze a range of chemical reactions, including cross-coupling [29], olefin metathesis [30], and hydrogenation reactions [31]. However, research on M–NHC functionalized coordination polymers in electrocatalysis remains limited. Introducing metal active sites via post-synthetic modification of imidazolium porous organic polymers and utilizing them for electrocatalytic water-splitting holds great significance.

Herein, a series of metal–*N*-heterocyclic carbene POPs were synthesized from imidazolium borate ionic POPs and supported on foam nickel (Pd–NHC/NF, Ag–NHC/NF and Cu–NHC/NF) as bifunctional electrocatalysts for electrocatalytic water-splitting. The POPs serve as multifunctional catalytic platforms in water-splitting reactions. Their porous architecture facilitates efficient mass transport and electrolyte accessibility, while the tunable coordination environment enables strong metal–ligand interactions that regulate the electronic structure of active sites. Such synergistic effects can optimize the adsorption energetics of key intermediates (*H, *OH, *O, *OOH), thereby enhancing HER and OER kinetics. In addition, the networks of POPs can stabilize dispersed metal species and prevent aggregation under electrochemical conditions, ensuring long-term catalytic durability. Among them, Pd–NHC/NF with Pd nanoparticles loaded in its matrix exhibited low overpotential of 245 mV (*η*_10_) for OER, and 139 mV (*η*_10_) for HER in alkaline medium. Additionally, the Pd–NHC/NF||Pd–NHC/NF electrolyzer demonstrated excellent activity, with a voltage of 1.55 V at 10 mA cm^−2^ and maintained high stability for overall water-splitting catalysis over a 90 h long-term (*E-t*) test.

## 2. Materials and Methods

### 2.1. Synthesis of BIM-ph

1,4-Bis(bromomethyl)benzene (151.0 mg, 0.50 mmol) was dissolved in 30 mL of CH_3_CN under N_2_ atmosphere and Na[B(IM)_4_] (307.8 mg, 1.17 mmol) was added. The mixture was heated to reflux for 48 h. The mixture was cooled to room temperature and subsequent filtration afforded a white precipitate. The precipitate was washed with acetonitrile (5 mL × 3) and EtOH (5 mL × 3) and then dried under vacuum to obtain a white solid (273.1 mg, 89%).

### 2.2. Synthesis of Pd–NHC

A reaction mixture of BIM-ph (33.2 mg, 0.04 mmol) and Pd(OAc)_2_ (36.0 mg, 0.16 mmol) in 25 mL of DMF was stirred and heated to reflux for 24 h under N_2_ atmosphere. After the reaction mixture was cooled to room temperature, the precipitate was collected by filtration, washed three times with acetone (5 mL × 3), and dried in a vacuum to obtain a dark grey solid (29.8 mg, 59%).

### 2.3. Synthesis of Ag–NHC

A mixture of BIM-ph (33.2 mg, 0.04 mmol) and Ag_2_O (19.0 mg, 0.08 mmol) was suspended in anhydrous acetonitrile (20 mL). The reaction mixture was stirred and heated at 70 °C under N_2_ atmosphere for 24 h in dark, protected from light. After cooling, the solid was filtered under anhydrous and oxygen-free conditions and washed with 10% NH_3_·H_2_O to remove residual Ag_2_O. The resulting precipitate was collected by filtration and washed with water (5 mL × 3) and EtOH (5 mL × 3) to obtain a grey solid (29.0 mg, 69%).

### 2.4. Synthesis of Cu–NHC

BIM-ph (33.2 mg, 0.04 mmoL) was dispersed in acetone under N_2_ atmosphere, and then CuCl (15.8 mg, 0.16 mmoL) and K_2_CO_3_ (44.2 mg, 0.32 mmoL) were added to the mixture. The resulting mixture was heated to reflux for 24 h. After cooling, the mixture was filtered under N_2_ protection and washed with acetone (5 mL × 3) and ethanol (5 mL × 3) to yield a yellow solid (16.7 mg, 39%).

### 2.5. Preparation of Pd–NHC/NF, Ag–NHC/NF, and Cu–NHC/NF

Foam nickel was treated by ultrasonication in acetone, distilled water, 0.2 mol L^−1^ NaBH_4_, and water for 30 min, and then dried at 70 °C. The preparation of the catalyst ink involved the dispersing of 5.0 mg Pd–NHC in a mixture of 145 μL of H_2_O, 335 μL of isopropanol, and 20 μL of nafion. The ink was subjected to ultrasonic treatment for 30 min to achieve a homogenized dispersion and then drop-casted onto the treated nickel foam to obtain Pd–NHC/NF; the loading mass of the catalyst was 1.2 mg cm^−2^.

Ag–NHC–NF and Cu–NHC–NF were prepared using the same method by replacing Pd–NHC with Ag–NHC and Cu–NHC, respectively.

### 2.6. Electrochemical Measurements

Electrochemical OER, HER, and water-splitting performance of the prepared catalysts were evaluated using an electrochemical workstation (CHI 660E, CHI Instruments, Shanghai, China) at room temperature (25 °C). The tests were conducted using a three-electrode system with an electrolyte of 1.0 mol L^−1^ KOH. The working electrode (Pd–NHC/NF, Ag–NHC/NF or Cu–NHC/NF) was prepared by coating Pd–NHC, Ag–NHC, or Cu–NHC onto nickel foam (NF, 0.25 cm^−2^), as shown above. A carbon rod with an area of 3.0 cm^−2^ was used as the counter electrode; a Hg/HgO electrode containing 1.0 mol L^−1^ KOH electrolyte was used as the reference electrode. The measured potential was referenced to the reversible hydrogen electrode (RHE) by applying the equation *E* (vs. RHE) = *E* (vs. Hg/HgO) + 0.059 × pH + 0.098. The scanning rate of LSV was 10 mV s^−1^. The Tafel slopes were determined by linearly fitting the experimental data using the Tafel equation, *η* = *a* + *b* log |*j*|, where *η*, *b,* and *j* correspond to overpotential, Tafel slope, and current density, respectively. EIS measurements were performed at 0.500 V for the OER and −0.132 V for the HER, with an AC voltage amplitude of 5 mV over a frequency range of 10^5^ Hz–0.01 Hz. The double-layer capacitance (*C*_dl_) at the solid–liquid interface was determined by cyclic voltammetry (CV) to estimate the ECSA. The measurements were carried out in the potential ranges of 1.10 to 1.20 V (vs. RHE) for OER and 0.10 to 0.20 V (vs. RHE) for HER to ensure negligible Faradic current on the working electrode. CV measurements were conducted at scanning rates of 20, 40, 60, 80, and 100 mV s^−1^. A 95% iR compensation was applied to all reported electrochemical data.

## 3. Results and Discussion

### 3.1. Preparation and Characterization

**Figure 1:**
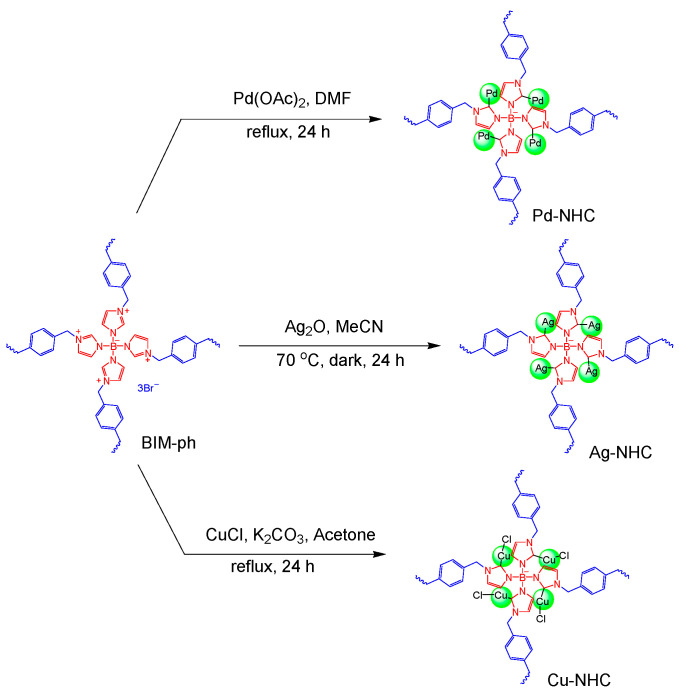
Synthesis of Pd–NHC, Ag–NHC, and Cu–NHC.

**Figure 2:**
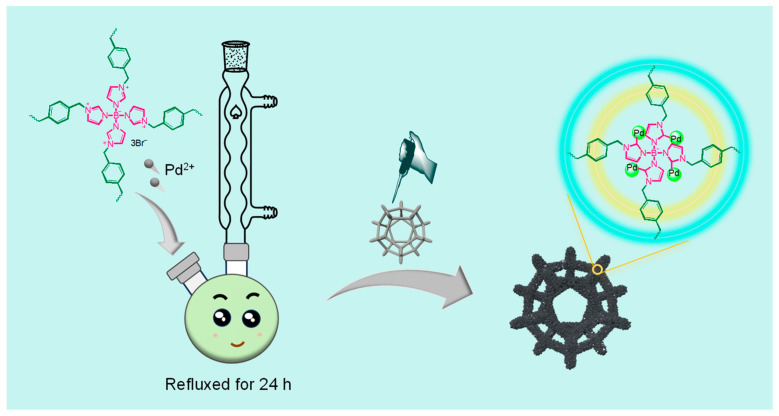
Schematic diagram of the fabrication process for Pd–NHC/NF.

Metal *N*-heterocyclic carbene polymers (Pd–NHC, Ag–NHC and Cu–NHC) were prepared from an imidazolium borate ionic POP (Figure 1). First, sodium tetrakis(1-imidazolyl) borate (Na[B(IM)_4_]) was obtained by heating the mixture of imidazole and NaBH_4_ at 225 °C under an N_2_ atmosphere, according to previously reported procedures [32]. Then, Na[B(IM)_4_] reacted with 1,4-bis(bromomethyl)benzene via a solvothermal method to form imidazolium borate ionic POP with Br^-^ as counterions (BIM-ph) [33]. BIM-ph is ionic POP with positively charged imidazolium groups and negatively charged borate groups. BIM-ph, as a precursor of NHC ligand, form stable metal–*N*-heterocyclic carbene polymers, Pd–NHC, Ag–NHC, and Cu–NHC, after complexation with Pd(OAc)_2_, Ag_2_O and CuCl (Figure 2). NHCs possess strong σ-donating ability and form stable M–NHC bonds upon coordination with metal ions (Pd^2+^, Ag^+^ and Cu^+^). The strong metal–carbene bonds endowed the metal–*N*-heterocyclic carbene polymers with excellent stability to air and moisture.

**Figure 3:**
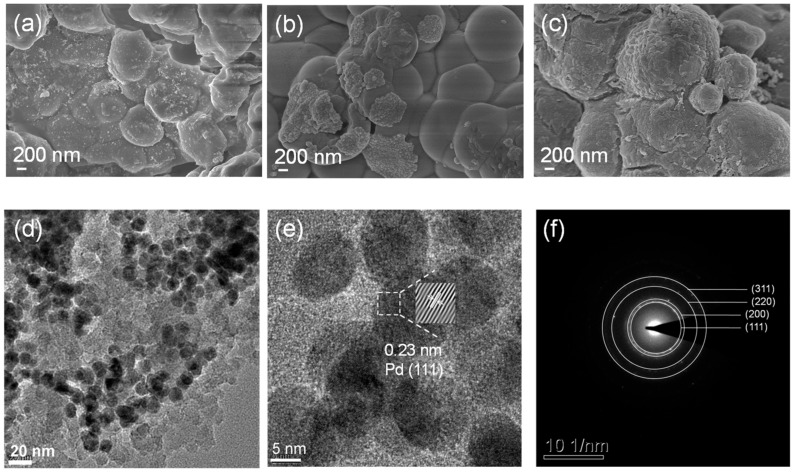
SEM images of: (**a**) Pd–NHC; (**b**) Ag–NHC; (**c**) Cu–NHC; (**d**) TEM image; (**e**) HR-TEM image; and (**f**) the corresponding SAED pattern of Pd–NHC.

Scanning electron microscopy (SEM) was used to study the morphology (Figure 3). SEM revealed that Pd–NHC consisted of aggregated spherical particles of 1–1.2 μm. Ag–NHC also possessed a porous structure with aggregated spherical particles, measuring approximately 1.2 μm in diameter. Similarly, Cu–NHC had a morphology of aggregated spherical particles with a diameter of 2–2.4 μm. The morphology of Pd–NHC were also investigated by high-resolution transmission electron microscopy (TEM). TEM images revealed uniform distribution of Pd NPs with particles sized 10 to 15 nm immobilized within the Pd–NHC matrix. HR-TEM images revealed distinct lattice fringes. The interplanar spacing of 0.23 nm is attributable to the (111) planes of metallic Pd [34]. Additionally, selected-area electron diffraction (SAED) exhibited a polycrystalline ring pattern, which is consistent with the diffraction planes of metallic Pd and the standard reference data for palladium (JCPDS Card No. 05-0681) [35]. Furthermore, the palladium content of Pd–NHC was determined to be 9.74 wt% using inductively coupled plasma–optical emission spectrometry (ICP–OES).

**Figure 4:**
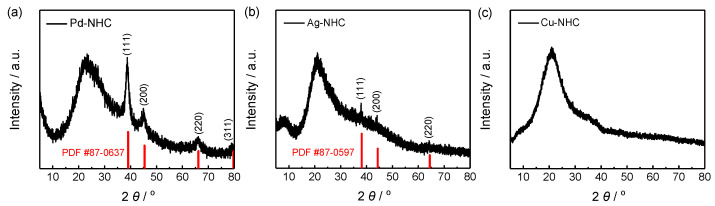
XRD patterns of: (**a**) Pd–NHC; (**b**) Ag–NHC; and (**c**) Cu–NHC.

Powder X-ray diffraction (PXRD) of Pd–NHC revealed diffraction peaks at 39.06°, 45.41°, 66.17° and 79.60°, which are assigned to the (111), (200), (220) and (311) planes of Pd metal, respectively (JCPDS Card No.05-0681) [35], indicating that Pd^2+^ may be reduced to Pd^0^ in DMF (Figure 4). Ag–NHC exhibited weak diffraction peaks at 38.12°, 44.28° and 64.20°, assignable to the (111), (200) and (220) crystallographic planes of Ag metal, respectively (JCPDS Card No.87-0597) [36], indicating that a small amount of Ag^+^ may have been reduced to Ag^0^. Cu–NHC showed a broad peak at about 21°, suggesting its amorphous nature.

**Figure 5:**
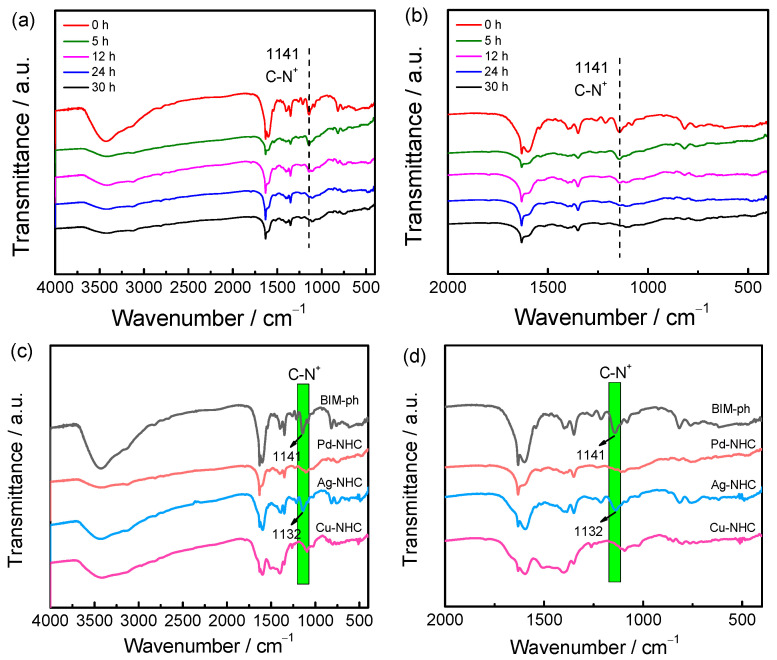
(**a**,**b**) Ex situ FT–IR spectra of Pd–NHC at different reaction times (0 h, 5 h, 12 h, 24 h and 30 h), and (**c**,**d**) FT–IR spectra of Pd–NHC, Ag–NHC, Cu–NHC and BIM-ph.

FT–IR spectroscopy was employed to monitor the formation of Pd–NHC at different reaction times (0, 5, 12, 24 and 30 h) (Figure 5). For BIM-ph, the band at 1141 cm^−1^ is attributed to the C–N^+^ stretching vibration of the imidazole ring [33,37,38,39]. The ex situ FT–IR spectra revealed gradual weakening of the imidazolium C–N^+^ bond as the reaction progressed, confirming the formation of metal–NHC complexes [40,41]. Similarly, the characteristic band at 1141 cm^−1^ corresponds to the imidazolium C–N^+^ stretching vibration for Cu–NHC, which was obviously weakened compared with BIM–ph, demonstrating the successful preparation of Cu–NHC polymers [42]. Additionally, the stretching frequency of the C–N^+^ bond decreases from 1141 cm^−1^ to 1132 cm^−1^ for Ag–NHC. The red shift is attributed to the metal–NHC bonding, probably resulting from charge delocalization within the particles due to reduced metal–ligand interfacial resistance [43].

**Figure 6:**
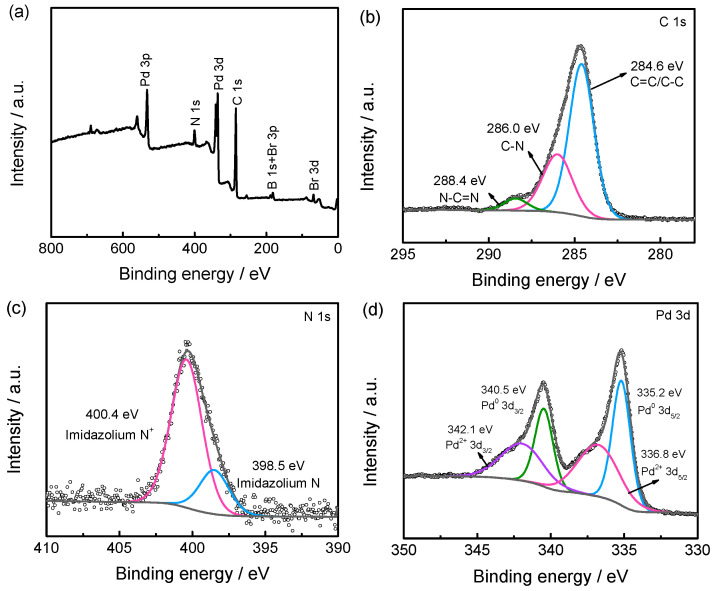
(**a**) XPS survey spectrum; (**b**) C 1s; (**c**) N 1s; and (**d**) Pd 3d deconvoluted high-resolution XPS spectra of Pd–NHC.

X-ray photoelectron spectroscopy (XPS) was employed to analyze the elements and determine the electronic properties of Pd–NHC (Figure 6). The high-resolution XPS C 1s spectrum consists of three deconvoluted peaks, which were assigned to C=C/C–C (284.6 eV), C=N (286.0 eV), and the sp^2^-hybridized carbon of the N–C=N group (288.4 eV) [44]. The resolution of the N 1s spectrum into two peaks suggests two chemically different types of nitrogen atoms, N–C (400.4 eV) and N=C (398.5 eV). The Pd 3d region consisted of two spin-orbit components of 3d_5/2_ and 3d_3/2_ [27]. Specifically, the chemical states of the palladium species in Pd–NHC were assessed. The Pd 3d region exhibited four distinct peaks of Pd^0^ and Pd^2+^, which were assigned to Pd^2+^ 3d_5/2_ at 336.8 eV, Pd^2+^ 3d_3/2_ at 342.1 eV, Pd^0^ 3d_5/2_ at 335.2 eV, and Pd^0^ 3d_3/2_ at 340.5 eV [25,45]. Compared with Pd(OAc)_2_, the binding energies of Pd^2+^ 3d_5/2_ and 3d_3/2_ in Pd–NHC showed negative shifts of 1.3 eV and 1.4 eV, respectively, demonstrating a strong chemical coordination interaction between Pd^2+^ and the *N*-heterocyclic carbene [46]. The presence of Pd^0^ indicated that a portion of Pd^2+^ was reduced during the coordination of the NHC ligand. As shown above, the Pd nanoparticles were also observed by XRD and TEM.

### 3.2. Performance of Oxygen Evolution Reaction

**Figure 7:**
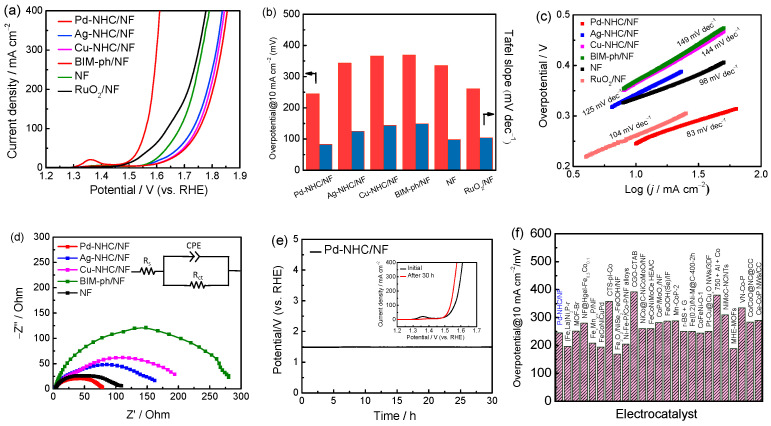
(**a**) LSV curses of Pd–NHC/NF, Ag–NHC/NF, Cu–NHC/NF, BIM-ph/NF, and RuO_2_/NF in 1.0 mol L^−1^ KOH for OER; (**b**) summary of overpotential and Tafel slopes as histograms for Pd–NHC/NF, Ag–NHC/NF, Cu–NHC/NF, BIM-ph/NF, and RuO_2_/NF; (**c**) Tafel curves of Pd–NHC/NF, Ag–NHC/NF, Cu–NHC/NF, BIM-ph/NF, and RuO_2_/NF in 1.0 mol L^−1^ KOH; (**d**) Nyquist plots of Pd–NHC/NF, Ag–NHC/NF, Cu–NHC/NF, BIM-ph/NF, and RuO_2_/NF in 1.0 mol L^−1^ KOH for OER (inset shows the equivalent circuit diagram); (**e**) chronopotentiometry *E-t* curve of Pd–NHC/NF (inset illustrates the polarization curves recorded initially and after 30 h); and (**f**) comparison of overpotentials at 10 mA cm^−2^ with recently reported OER electrocatalysts.

The OER catalytic activity of Pd–NHC, Ag–NHC and Cu–NHC, deposited on an NF electrode, was investigated with a sweep rate of 10 mV s^−1^ in 1.0 mol L^−1^ KOH as the electrolyte (Figure 7). BIM-ph/NF and RuO_2_/NF were studied as the control. A linear scanning voltammetry (LSV) test was carried out on the electrocatalysts. Pd–NHC/NF exhibited the highest activity for OER among all electrocatalysts, with the overpotential of 245 mV at a current density of 10 mA cm^−2^, surpassing Ag–NHC/NF (343 mV), Cu–NHC/NF (366 mV), BIM-ph/NF (369 mV), and commercial RuO_2_/NF (261 mV). By measuring the overpotentials at different current densities, the OER performance was further evaluated. The overpotentials required for Pd–NHC/NF to reach 100, 200, and 300 mA cm^−2^ were 331, 356 and 370 mV, respectively. In comparison, the overpotentials required for Ag–NHC/NF, Cu–NHC/NF, BIM-ph/NF, and RuO_2_/NF to reach 100 mA cm^−2^ were 501, 515, 524, and 408 mV, respectively, further demonstrating the superior OER performance of Pd–NHC/NF. Additionally, the mass activity of Pd–NHC was determined to be high, up to 85.56 mA mg^−1^ at the overpotential of 245 mV.

The Tafel slope was determined to investigate the kinetic characteristics of electrocatalytic reactions. Pd–NHC/NF showed the Tafel slope of 83 mV dec^−1^, significantly smaller than Ag–NHC/NF (125 mV dec^−1^), Cu–NHC/NF (144 mV dec^−1^), BIM-ph/NF (149 mV dec^−1^), and RuO_2_/NF (104 mV dec^−1^). It indicates that Pd–NHC/NF has higher activity, as the OER process rate increases more significantly with increasing potential.

The OER process involves a complex four-electron transfer through a multi-step mechanism; the formation of each oxygen molecule requires the transfer of four electrons. The OER process in alkaline media involves three reaction intermediates, NHC–M–OH, NHC–M–O, and NHC–M–OOH, where NHC–M is the active metal center of the electrocatalysts [47,48]. The intermediates represent adsorbed oxygen species attached to the active metal center of M–NHC polymer. Typically, under electrochemical oxidation conditions, hydroxyl ions first initiate the formation of NHC–M–OH, followed by deprotonation and oxidation steps to form the NHC–M–O and NHC–M–OOH intermediates, culminating in the release of O_2_.

Electrochemical impedance spectroscopy (EIS) was used to further investigate the charge transfer resistance and kinetic behavior of Pd–NHC/NF, Ag–NHC/NF and Cu–NHC/NF in alkaline media with a testing frequency range of 0.01–10^5^ Hz. EIS serves to elucidate the mechanism at electrode–electrolyte interfaces, where the components in the equivalent electrochemical circuit (EEC) correspond to their characteristics, solution resistance (*R*_s_), double-layer capacitance (CPE), and charge transfer resistance (*R*_ct_). The EIS results show that the *R*_ct_ of Pd–NHC/NF was 71.3 Ω, significantly lower than those of Ag–NHC/NF (148.3 Ω), Cu–NHC/NF (196.3 Ω), and BIM-ph/NF (279.2 Ω) (Figure 7d). The charge transfer impedance follows the order of Pd–NHC< Ag–NHC< Cu–NHC< BIM-ph. Among them, Pd–NHC has the smallest charge transfer resistance, indicating that it has the fastest electron transfer rate. Therefore, the favorable OER kinetics observed for Pd–NHC/NF may be attributed to the enhanced charge transfer ability [19,47,49].

A long-term durability test of Pd–NHC was conducted at 10 mA cm^−2^ for 30 h, during which it maintained a stable current density (Figure 7e). LSV indicates that the potential of Pd–NHC/NF had no significant change before and after the 30–h stability evaluation at 10 mA cm^−2^. Moreover, the potential decreased by only 1.17% at 50 mA cm^−2^, further demonstrating the excellent cycling stability of Pd–NHC/NF for OER.

To study the intrinsic OER electrocatalytic activity of the catalysts, cyclic voltammetry (CV) curves were recorded in the voltage range of 1.10–1.20 V (vs. RHE) (Figure S1). The electrochemical active area (ECSA) was evaluated by measuring the double-layer capacitance (*C*_dl_). The *C*_dl_ of Pd–NHC/NF (8.2 mF cm^−2^) exceeded those of Ag–NHC/NF (5.6 mF cm^−2^), Cu–NHC/NF (5.2 mF cm^−2^), and BIM-ph/NF (3.2 mF cm^−2^). It indicates that Pd–NHC/NF possessed a larger electrochemically active area and higher density of active sites [50,51], in agreement with its excellent OER performance.

The activity of Pd–NHC/NF is superior to that of many previously reported OER catalysts, including POPs (Figure 7f, Table S1) [51,52,53,54,55,56,57,58,59,60,61,62,63,64,65,66,67,68,69,70,71,72,73,74]. In comparison, NF@Hgel-Fe_0.3_Co_0.1_ (hydrogel containing Fe and Co) demonstrated an overpotential of 280 mV at a current density of 10 mA cm^−2^ [52], THT-PyDAN (C=C bond-linked POP containing nitrogen and sulfur active sites) possessed an overpotential of 283 mV at 10 mA cm^−2^ [53], TDA-Trz-POP (nitrogen/sulfur-rich thiadiazole- and triazine-linked POP) revealed an overpotential of 410 mV at 10 mA cm^−2^ [54], and Au(I)–NHC dinuclear complex exhibited an OER overpotential of 1140 mV in 1.0 mol L^−1^ KOH [55]. The present study provides an innovative approach for boosting OER performance.

### 3.3. Performance of Hydrogen Evolution Reaction

**Figure 8:**
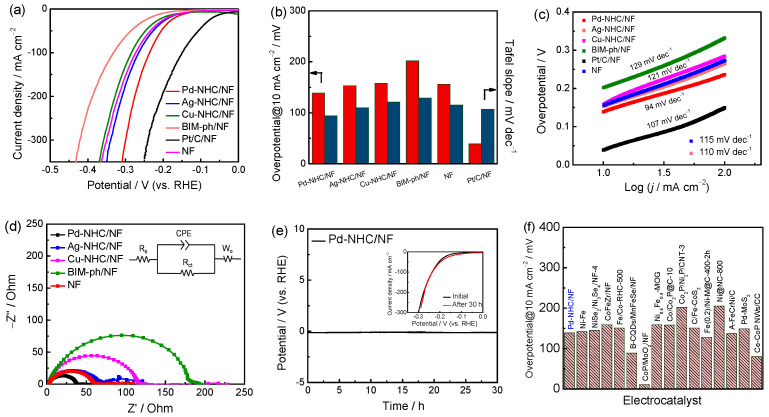
(**a**) LSV curses of Pd–NHC/NF, Ag–NHC/NF, Cu–NHC/NF, BIM-ph/NF, and Pt/C/NF in 1.0 mol L^−1^ KOH for HER; (**b**) histograms of overpotential and Tafel slopes for Pd–NHC/NF, Ag–NHC/NF, Cu–NHC/NF, BIM-ph/NF, and Pt/C/NF; (**c**) Tafel curves of Pd–NHC/NF, Ag–NHC/NF, Cu–NHC/NF, BIM-ph/NF, and Pt/C/NF in 1.0 mol L^−1^ KOH; (**d**) Nyquist plots of Pd–NHC/NF, Ag–NHC/NF, Cu–NHC/NF, BIM-ph/NF, and Pt/C/NF in 1.0 mol L^−1^ KOH for HER (inset shows the equivalent circuit diagram); (**e**) *E-t* curve of Pd–NHC/NF (inset presents the corresponding polarization curves before and after chronopotentiometry measurements for 30 h); and (**f**) comparison of overpotentials at 10 mA cm^−2^ with recently reported HER electrocatalysts.

The HER performance of Pd–NHC, Ag–NHC, and Cu–NHC, deposited on an NF electrode, was studied in 1.0 mol L^−1^ KOH (Figure 8). According to the LSV test, the overpotential of Pd–NHC/NF (139 mV) was significantly lower than that of Ag–NHC/NF (153 mV), Cu–NHC/NF (158 mV), and BIM-ph/NF (202 mV) at 10 mA cm^−2^, suggesting that Pd–NHC/NF was more effective and needed less energy to promote HER reactions. The outstanding HER activity of Pd–NHC/NF may arise from the strong σ-donor ability of the NHC ligand, which forms strong Pd-C bonds with Pd^2+^ and exposes more active sites by the metal ions [47]. Furthermore, Pd–NHC/NF has a smaller Tafel slope, of 94 mV dec^−1^, than Ag–NHC/NF (110 mV dec^−1^), Cu–NHC/NF (121 mV dec^−1^), and BIM-ph/NF (129 mV dec^−1^). As the control, commercial Pt/C/NF exhibited an overpotential of 39 mV at 10 mA cm^−2^ with a Tafel slope of 107 mV dec^−1^. The Tafel slope of Pd–NHC/NF may arise from the Volmer–Heyrovsky mechanism of hydrogen electrochemical desorption, with the Heyrovsky step being the possible rate-determining step [7,75].

The electrode kinetics of the catalyst was studied through EIS measurement. The charge transfer resistance of Pd–NHC/NF (37.2 Ω) was lower than that of Ag–NHC/NF (67.8 Ω), Cu–NHC/NF (116.2 Ω), and BIM-ph/NF (179.0 Ω), indicating faster chemical reaction kinetics (Figure 8d). The long-term durability performance of Pd–NHC/NF was evaluated for 30 h at 10 mA cm^−2^ (Figure 8e). The LSV curves of Pd–NHC/NF before and after long-term stability testing were nearly superimposed, confirming its excellent durability.

The ECSA was evaluated based on the *C*_dl_ measured via CV. In Figure S2, Pd–NHC/NF exhibited the highest *C*_dl_ (1.5 mF cm^−2^), which is superior to those of Ag–NHC/NF (1.2 mF cm^−2^), Cu–NHC/NF (0.6 mF cm^−2^), and BIM-ph/NF (0.5 mF cm^−2^), indicating that Pd–NHC/NF had higher ECSA, meaning that it has a larger catalytic active area and more active sites.

Pd–NHC/NF exhibits competitive HER performance, which is comparable to many advanced electrocatalysts, including POPs (Figure 8f, Table S2) [76,77,78,79,80,81,82,83,84,85,86,87]. For example, Ni@COP (nickel-doped 2D covalent organic polymer) exhibited an overpotential (*η*_10_) of 290 mV and a Tafel slope of 112 mV dec^−1^ in 0.5mol L^−1^ H_2_SO_4_ [76], TPA-OMe (triphenylamine-based POP) demonstrated an overpotential (*η*_10_) of 353 mV in 0.5 mol L^−1^ H_2_SO_4_ [77], and SMCOP-4 (triazine-containing imine POP) indicated a HER overpotential of 139 mV at 10 mA cm^−2^ [78].

### 3.4. Performance of Overall Water-Splitting

**Figure 9:**
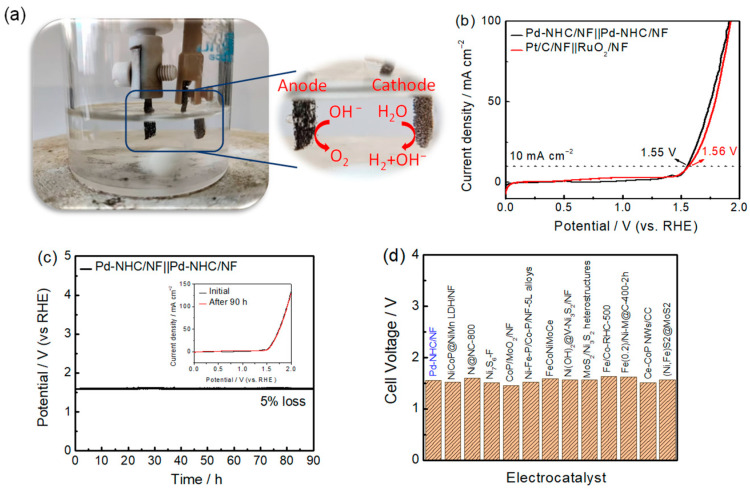
(**a**) Digital photograph of the Pd–NHC/NF||Pd–NHC/NF electrolyzer; (**b**) linear sweep voltammetric curves of Pd–NHC/NF(+)||Pd–NHC/NF(−) in the two-electrode system; (**c**) stability of Pd–NHC/NF(+)||Pd–NHC/NF(−) at 10 mA cm^−2^ (inset shows the LSV curves recorded initially and after 90 h of testing); and (**d**) cell voltage to reach 10 mA cm^−2^ with reported catalysts for the overall water-splitting performance in 1.0 mol L^−1^ KOH.

The above investigations indicate that the Pd–NHC/NF electrode exhibited excellent OER and HER activity and can be used as a bifunctional electrocatalyst. Pd–NHC/NF was subsequently used as anode and cathode in a two-electrode configuration to explore the overall water-splitting performance (Figure 9a). The Pd–NHC/NF||Pd–NHC/NF electrolyzer required a voltage of 1.55 V to achieve a current density of 10 mA cm^−2^ at in 1.0 mol L^−1^ KOH (Figure 9b), which is better than that of the benchmark Pt/C/NF||RuO_2_/NF (1.56 V) at 10 mA cm^−2^. For Pt/C/NF||RuO_2_/NF, commercial Pt/C and RuO_2_ were used as cathode and anode, respectively. Additionally, chronopotentiometry tests on the Pd–NHC/NF||Pd–NHC/NF electrode for overall water decomposition showed that the potential only increased by 5% after 90 h of electrolysis at a constant current density of 10 mA cm^−2^, demonstrating excellent stability (Figure 9c). The nearly overlapping LSV curves before and after the tests further confirmed its outstanding durability. Interestingly, the water-splitting activity of Pd–NHC/NF||Pd–NHC/NF surpasses and/or matches that of advanced catalysts reported in the literature for electrocatalytic water-splitting [17,88,89,90,91,92,93] such as NiTAPP–NiACQ (porphyrin POP cathode with a cell voltage of 1.59 V at 10 mA cm^−2^) [88], MoS_2_/Ni_3_S_2_ (heterostructure with a cell voltage of approximately 1.56 V at 10 mA cm^−2^) [89], Fe(0.2)/Ni-M@C-400-2h (iron-doped annealed Ni–MOF composite with a cell voltage of 1.62 V at 10 mA cm^−2^) [17], and so on (Figure 9d and Table S3).

Further characterization was conducted to investigate the structural integrity of Pd–NHC after the long-term stability test using TEM, FT–IR, and XRD (Figure S3). TEM images indicated that the structure did not change. Pd NPs with a size distribution of 10–15 nm were uniformly dispersed within the Pd–NHC matrix.HR-TEM analysis showed lattice fringes with an interplanar spacing of 0.23 nm. Additionally, the SAED pattern obtained from the Pd–NHC region further confirmed its crystalline structure. In the FT–IR spectra, the band at 1630 cm^−1^ and 1141 cm^−1^ was assigned to the stretching vibrations of the C=N bond and the C-N^+^ bond within the imidazole ring, respectively. The XRD pattern of the spent Pd–NHC catalyst showed diffraction peaks at 2*θ* = 39.06°, 45.41°, 66.17° and 79.60°, which were assignable to the (111), (200), (220) and (311) Bragg reflections of metallic Pd (Pd^0^), respectively. This is consistent with that of the fresh catalyst, demonstrating that the Pd–NHC catalyst had excellent structural stability under the reaction conditions. The structural characteristics of Pd–NHC remained unchanged after electrocatalysis, suggesting that the material possesses excellent stability for electrocatalytic water-splitting.

To quantitatively assess the number and efficiency of active sites and provide further evidence for the superior electrocatalytic performance of Pd–NHC, turnover frequency (*TOF*) was determined in alkali electrolytes [4,94]. *TOF* was calculated using the equation, TOF=jA/(zFn), where *j* is the measured current density at a specified overpotential, *A* is the geometric area of the NF working electrode (0.25 cm^−2^ in this work), *z*denotes the amount of active sites available for catalysis, *F* is the Faraday constant (96,485 C mol^−1^), and *n* equals 4 for the OER or 2 for the HER. Pd–NHC showed OER activity with an overpotential of 245 mV to achieve 10 mA cm^−2^, corresponding to TOF_OER_ of 0.024 s^−1^. Similarly, Pd–NHC required an overpotential of 139 mV to achieve 10 mA cm^−2^ for the HER, yielding TOF_HER_ of 0.047 s^−1^. The remarkable electrocatalytic activity may be ascribed to the catalyst’s abundance of highly concentrated active sites, which facilitate the efficient adsorption and desorption of H^+^ and OH^−^ ions.

## 4. Conclusions

To sum up, a series of metal–nitrogen heterocyclic carbenes supported on nickel foam (Pd–NHC/NF, Ag–NHC/NF, and Cu–NHC/NF) were developed as bifunctional electrocatalysts for efficient water electrolysis. Metal–nitrogen heterocyclic carbenes (M–NHCs) were synthesized from imidazolium borate ionic POP and metal ions (Pd^2+^, Ag^+^, and Cu^+^). The utilization of imidazolium borate ionic POP as NHC ligand leads to strongly bound M–NHC species with metal ions, facilitated by the strong electron-donating character of the NHCs. Among the materials, Pd–NHC with Pd NPs loaded in its networked matrix demonstrated superior performance in both the HER and the OER. To achieve a current density of 10 mA cm^−2^ in alkaline medium, Pd–NHC/NF exhibited a low OER overpotential of 245 mV in alkaline medium with a Tafel slope of 83 mV dec^−1^, and a low overpotential of 139 mV in HER with a Tafel slope of 94 mV dec^−1^. Moreover, Pd–NHC also demonstrated outstanding overall water-splitting activity (1.55 V at *η*_10_) on NF and exhibited exceptional durability, with no noticeable decay during a continuous 90-h *E-t* curve in alkaline medium. Overall, this work presents an efficient bifunctional electrocatalyst for efficient water-splitting.

## Data Availability

The original contributions presented in this study are included in the article. Further inquiries can be directed to the corresponding author.

## Supplementary Materials

The following supporting information can be downloaded at: https://www.mdpi.com/article/10.3390/nano16120781/s1. Additional experimental details, electrocatalysis and characterization data. Table S1. Comparison of OER performance of Pd-NHC with those of reported electrocatalysts. Table S2. Comparison of HER performance of Pd-NHC with those of reported electrocatalysts. Table S3. Comparison of the overall water splitting performance with previously reported comparable electrocatalysts as electrolyzers. Figure S1. (a) CV curves of Pd-NHC/NF at various scan rates, (b) CV curves of Ag-NHC/NF at various scan rates, (c) CV curves of Cu-NHC/NF at various scan rates, (d) CV curves of BIM-ph/NF at various scan rates, (e) CV curves of RuO2/NF at various scan rates, (f) plots of ΔJ versus scan rate for Pd-NHC/NF, Ag-NHC/NF, Cu-NHC/NF, BIM-ph/NF and RuO2/NF at various scan rates. Figure S2. (a) CV curves of Pd-NHC/NF at various scan rates; (b) CV curves of Ag-NHC/NF at various scan rates; (c) CV curves of Cu-NHC/NF at various scan rates; (d) CV curves of BIM-ph/NF at various scan rates; (e) CV curves of Pt/C/NF at various scan rates; (f) plots of ΔJ versus scan rate for Pd-NHC/NF, Ag-NHC/NF, Cu-NHC/NF, BIM-ph/NF and Pt/C/NF at various scan rates. Figure S3. (a) TEM image, (b) HR-TEM image, and (c) the corresponding SAED pattern of Pd-NHC after long-term water splitting for 90 h. (d) FT-IR spectra and (e) powder XRD patterns of Pd-NHC before and after long-term water splitting for 90 h.
