# Supplementary material for: Metal–N-Heterocyclic Carbene Porous Organic Polymers as Efficient Bifunctional Water-Splitting Electrocatalysts
Source: Nanomaterials (Basel). 2026 Jun 21;16(12):781. doi: 10.3390/nano16120781 (PMC13305236; doi:10.3390/nano16120781)
Supplement: Supplementary file 1 [file nanomaterials-16-00781-s001.zip › nanomaterials-4378410-supplementary.pdf]

Supplementary material

Metal-*N*-heterocyclic carbene porous organic polymers as efficient bifunctional water-splitting electrocatalysts

Shasha Ma [a], Zhaobin Ye [a], Guang Shi [b] and Jianyong Zhang [a,*]

[a] *Sun Yat-Sen University, MOE Laboratory of Polymeric Composite and Functional Materials, School of Materials Science and Engineering, Guangzhou 510275, China.*

[b] *School of Chemistry, South China Normal University, Guangzhou 510006, China.*

E-mail: zhjyong@mail.sysu.edu.cn (J. Z.)

Experimental section

Materials and characterization

All reagents and solvents were purchased from commercial suppliers and used as received without further purification unless otherwise stated. All experiments were performed under nitrogen atmosphere using standard Schlenk techniques. Solvents were degassed and distilled under nitrogen atmosphere from CaH_2 (DMF and CH_3CN) prior to use. FT-IR spectra were recorded on a Thermo Fisher Nicolet iS10 FT-IR spectrometer with KBr pellets in the range 4000-400 cm^{-1}. ^{1}H NMR spectra were recorded on a Bruker Avance III/400 (400 MHz) spectrometer. Scanning electron micrograph (SEM) was recorded on a Gemini500 emission environmental scanning electron microscope. Samples were prepared by dispersing in anhydrous ethanol upon sonication and placing on top of aluminum foil. Transmission electron microscopy (TEM) was conducted on a FEI Tecnai G2 Spirit 300 kV transmission electron microscope. Samples were prepared by dispersing in anhydrous ethanol upon sonication and placing on top of the carbon grid. Metal analyses were performed by using an Optima 8300 ICP-OES instrument. Before analysis, the material was digested by aqua regia at 80 ^{o}C for 12 h. X-ray photoelectron spectroscopy (XPS) was tested on NexsaThermo ESCALAB 250Xi instrument with an Al Kα X-ray source (Thermo Fisher). Power X-ray diffraction (PXRD) measurement was performed on D-MAX 2200 VPC instrument.

Table S1. Comparison of OER performance of Pd-NHC with those of reported electrocatalysts.

Catalyst	Substrate	electrolyte	Current density / mA cm^{-2}	Overpotential / mV	Tafel slope / mV dec^{-1}	Ref
Pd-NHC	NF	1.0 M KOH	10	245	83	This work
THT-PyDAN	Carbon cloth	1.0 M KOH	10	283	81	53
Au(I)-NHC	GCE	1.0 M KOH	10	1140	30.4	55
TDA-Trz-POP	graphite carbon	1.0 M KOH	10	410	104.5	54
(Fe,La)Ni_2P-r	NF	1.0 M KOH	10	197	30	56
MOF-Br	NF	1.0 M KOH	10	251.2	44.5	57
NF@Hgel-$Fe_{0.3}Co_{0.1}$	NF	1.0 M KOH	10	280	78	52
$Fe_xMn_{y-x}P$/NF	NF	1.0 M KOH	10	208	32	58
FeCoNiCuPd	NF	1.0 M KOH	10	194	39.8	59
CTS-pl-Co	NF	0.1 M KOH	10	358	76	60
Fe_2O_3/$NiSe_2$-FeOOH/NF	NF	1.0 M KOH	10	169	31.2	61
Ni-Fe-P/Co-P/NF alloys	NF	1.0 M KOH	10	205	49.6	51
CGO-CTAB	NF	1.0 M KOH	10	391.5	56.4	62
NiCo@C-NiCoMoO/NF	NF	1.0 M KOH	10	260	75.15	63
FeCoNiMoCe HEA/C	NF	1.0 M KOH	10	260	63.4	64
CoP/MoO_2/NF	NF	1.0 M KOH	10	282	36.2	19
FeOOH(Se)/IF	IF	1.0 M KOH	10	287	54	65
Mn-CoP-2	GCE	1.0 M KOH	10	288	77.2	66
r-BS + G	GCE	1.0 M KOH	10	250	210	67
Fe(0.2)/Ni-M@C-400-2h	GCE	1.0 M KOH	10	250	43.4	17
CoFeNi-O-1	GCE	1.0 M KOH	10	244	55.4	68
Pt-Cu@CuxO NWs/3DF	RDE	0.1 M KOH	10	250	117	7
750 + Al + Co	graphite foil	1.0 M KOH	10	380	91	69
NiMoC-NCNTs	Carbon paper	1.0 M KOH	10	310	63.5	70
MHE-MOFs	Carbon cloth	1.0 M KOH	10	189	41.5	71
VN-Co-P	Carbon cloth	1.0 M KOH	10	335	—	72
Co/CoO@NC@CC	Carbon cloth	1.0 M KOH	10	284	76	73
Ce-CoP NWs/CC	Carbon cloth	1.0 M KOH	10	290	152.7	74

Table S2. Comparison of HER performance of Pd-NHC with those of reported electrocatalysts.

Catalyst	Substrate	electrolyte	Current density/ mA cm^{-2}	Overpotential / mV	Tafel slope / mV dec^{-1}	Ref
Pd-NHC	NF	1.0 M KOH	10	139	94	This work
TPA-OMe	GCE	0.5 M H_2SO_4	10	353	130	77
Ni@COP	carbon paper	0.5 M H_2SO_4	10	290	112	76
SMCOP-4	NF	0.5 M KOH	10	139	133	78
Ni-Fe	NF	1.0 M KOH	10	142	133.3	79
$NiSe_2/Ni_3Se_4$/NF-4	NF	1.0 M KOH	10	145	69.7	80
CoFeZr/NF	NF	1.0 M KOH	10	159	132.7	81
Fe/Co-RHC-500	Nickel sheet	1.0 M KOH	10	151	63.4	22
B-CQDs/MnFeSe/NF	NF	1.0 M KOH	10	89	99.69	82
CoP/MoO_2/NF	NF	1.0 M KOH	10	11	83.6	19
$Ni_{0.6}Fe_{0.4}$-MOG	NF	1.0 M KOH	10	159	—	83
Co/Co_2P@C-10	GCE	1.0 M KOH	10	158	64.42	6
Co_2P/Ni_2P/CNT-3	GCE	1.0 M KOH	10	202	57.95	84
C/Fe-CoS_2	GCE	0.5 M H_2SO_4	10	151	66.21	85
Fe(0.2)/Ni-M@C-400-2h	GCE	1.0 M KOH	10	128	68.0	17
Ni@NC-800	GCE	1.0 M KOH	10	205	160	86
A-FeCrNi/C	RDE	1.0 M KOH	10	137	130.7	87
Pd-MoS_2	carbon paper	1.0 M KOH	10	149	72	16
Ce-CoP NWs/CC	Carbon cloth	1.0 M KOH	10	80	68.1	74

Table S3. Comparison of the overall water splitting performance with previously reported comparable electrocatalysts as electrolyzers.

Catalyst	Substrate	Electrolyte	Cell voltage / V	Ref
Pd-NHC	NF	1.0 M KOH	1.55 V@10 mA cm^{-2}	This work
NiCoP@NiMn LDH/NF	NF	1.0 M KOH	1.519 V@10 mA cm^{-2}	20
Ni@NC-800	NF	1.0 M KOH	1.60 V@10 mA cm^{-2}	86
Ni_7S_6-F	—	0.1 M KOH	1.51 V@10 mA cm^{-2}	1
CoP/MoO_2/NF	NF	1.0 M KOH	1.46 V@10 mA cm^{-2}	19
Ni-Fe-P/Co-P/NF-5L alloys	NF	1.0 M KOH	1.52 V@10 mA cm^{-2}	51
FeCoNiMoCe	NF	1.0 M KOH	1.59 V@10 mA cm^{-2}	64
$Ni(OH)_2$@V-Ni_3S_2/NF	NF	1.0 M KOH	1.57 V@10 mA cm^{-2}	18
MoS_2/Ni_3S_2 heterostructures	NF	1.0 M KOH	~1.56 V@10 mA cm^{-2}	89
Fe/Co-RHC-500	Nickel sheet	1.0 M KOH	1.63 V@10 mA cm^{-2}	22
Fe(0.2)/Ni-M@C-400-2h	carbon cloth	1.0 M KOH	1.62 V@10 mA cm^{-2}	17
Ce-CoP NWs/CC	Carbon cloth	1.0 M KOH	1.506 V@10 mA cm^{-2}	74
NiTAPP-NiACQ	NF	1.0 M KOH	1.59 V@10 mA cm^{-2}	88
Co-W-B-P/CF	carbon fiber	1.0 M KOH	1.57 V@10 mA cm^{-2}	90
MoS_2-Au	glassy carbon	0.5 M KOH	1.60 V@10 mA cm^{-2}	91
Pt-NiSe@NiFe-LDH-Ov	—	1.0 M KOH	1.878 V@50 mA cm^{-2}	92
(Ni, Fe)S_2@MoS_2	carbon fiber paper	1.0 M KOH	1.56 V@10 mA cm^{-2}	93

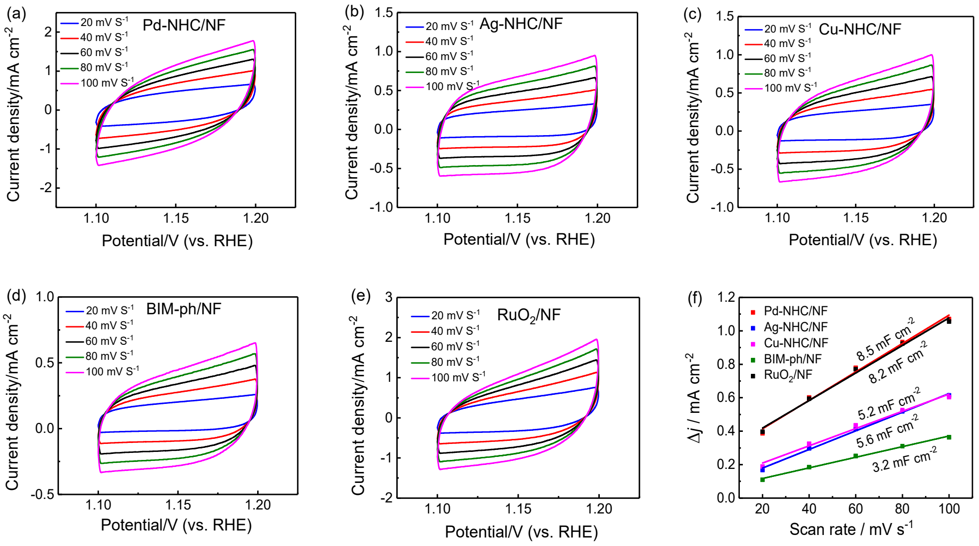

Figure S1. (a) CV curves of Pd-NHC/NF at various scan rates, (b) CV curves of Ag-NHC/NF at various scan rates, (c) CV curves of Cu-NHC/NF at various scan rates, (d) CV curves of BIM-ph/NF at various scan rates, (e) CV curves of RuO_2/NF at various scan rates, (f) plots of Δ*J* versus scan rate for Pd-NHC/NF, Ag-NHC/NF, Cu-NHC/NF, BIM-ph/NF and RuO_2/NF at various scan rates.

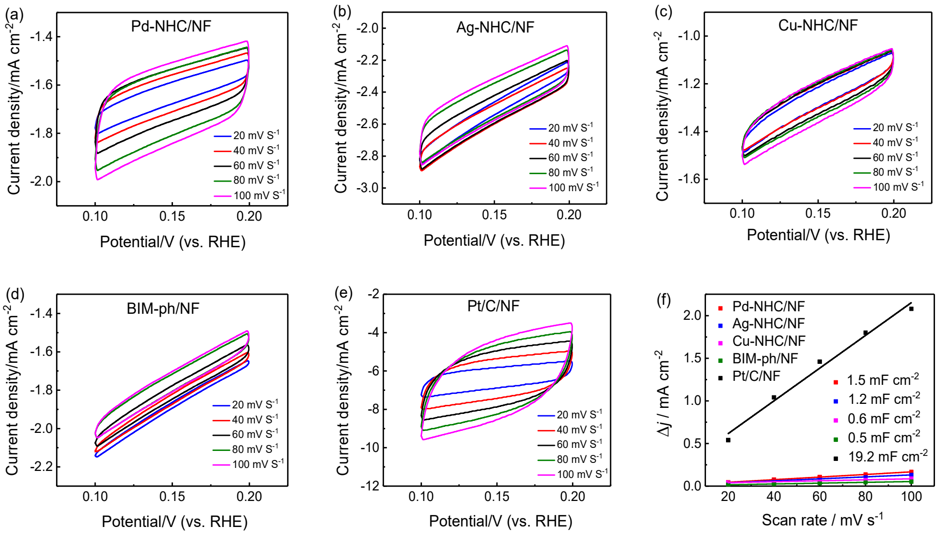

Figure S2. (a) CV curves of Pd-NHC/NF at various scan rates; (b) CV curves of Ag-NHC/NF at various scan rates; (c) CV curves of Cu-NHC/NF at various scan rates; (d) CV curves of BIM-ph/NF at various scan rates; (e) CV curves of Pt/C/NF at various scan rates; (f) plots of Δ*J* versus scan rate for Pd-NHC/NF, Ag-NHC/NF, Cu-NHC/NF, BIM-ph/NF and Pt/C/NF at various scan rates.

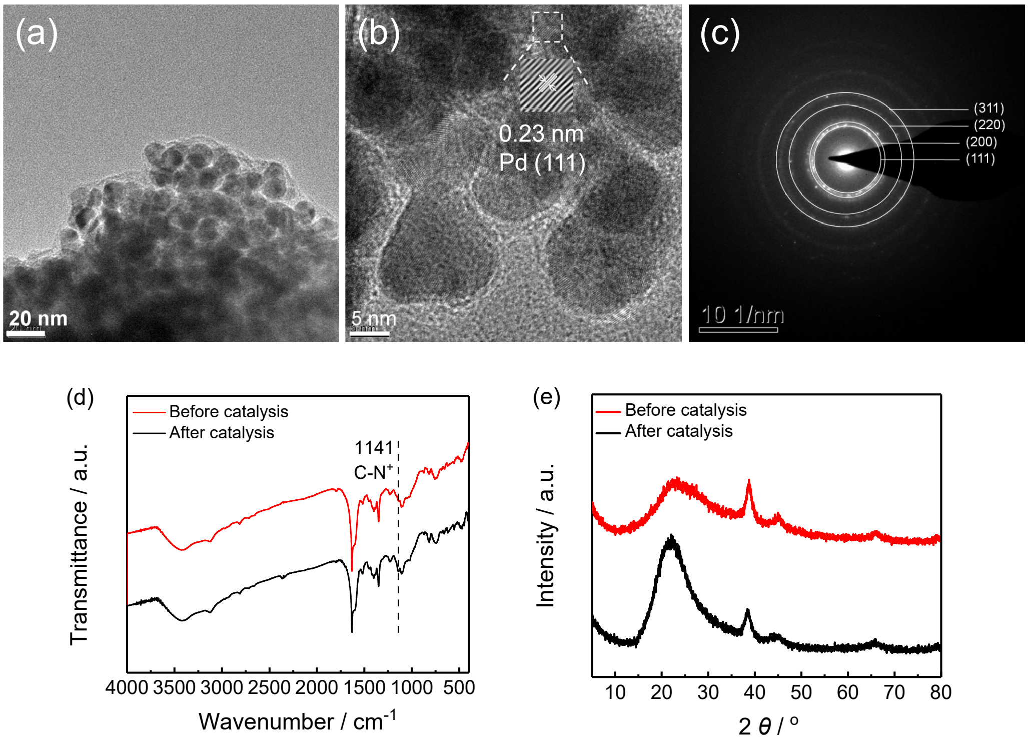

Figure S3. (a) TEM image, (b) HR-TEM image, and (c) the corresponding SAED pattern of Pd-NHC after long-term water splitting for 90 h. (d) FT-IR spectra and (e) powder XRD patterns of Pd-NHC before and after long-term water splitting for 90 h.
